# Therapeutic Effects of Dietary Soybean Genistein on Triple-Negative Breast Cancer via Regulation of Epigenetic Mechanisms

**DOI:** 10.3390/nu13113944

**Published:** 2021-11-04

**Authors:** Manvi Sharma, Itika Arora, Min Chen, Huixin Wu, Michael R. Crowley, Trygve O. Tollefsbol, Yuanyuan Li

**Affiliations:** 1 Department of Biology, University of Alabama at Birmingham, Birmingham, AL 35294, USA; manvi@uab.edu (M.S.); itiarora@uab.com (I.A.); huixin3@uab.edu (H.W.); 2 Department of Pharmacology and Toxicology, University of Alabama at Birmingham, Birmingham, AL 35294, USA; mchen117@uab.edu; 3 Department of Genetics, University of Alabama at Birmingham, Birmingham, AL 35294, USA; mcrowley@uab.edu; 4 O’Neal Comprehensive Cancer Center, University of Alabama at Birmingham, Birmingham, AL 35294, USA; 5 Integrative Center for Aging Research, University of Alabama at Birmingham, Birmingham, AL 35294, USA; 6 Nutrition Obesity Research Center, University of Alabama at Birmingham, Birmingham, AL 35294, USA; 7 Comprehensive Diabetes Center, University of Alabama at Birmingham, Birmingham, AL 35294, USA; 8 Department of Obstetrics, Gynecology & Women’s Heath, University of Missouri, Columbia, MO 65211, USA; 9 Department of Surgery, University of Missouri, Columbia, MO 65211, USA

**Keywords:** genistein, triple-negative breast cancer (TNBC), patient-derived xenograft (PDX), epigenetic, RNA-seq, cancer therapy

## Abstract

Consumption of dietary natural components such as genistein (GE) found in soy-rich sources is strongly associated with a lower risk of breast cancer. However, bioactive dietary component-based therapeutic strategies are largely understudied in breast cancer treatment. Our investigation sought to elucidate the potential mechanisms linking bioactive dietary GE to its breast cancer chemotherapeutic potential in a special subtype of aggressive breast cancer—triple-negative breast cancer (TNBC)—by utilizing two preclinical patient-derived xenograft (PDX) orthotopic mouse models: BCM-3204 and TM00091. Our study revealed that administration of GE resulted in a delay of tumor growth in both PDX models. With transcriptomics analyses in TNBC tumors isolated from BCM-3204 PDXs, we found that dietary soybean GE significantly influenced multiple tumor-regulated gene expressions. Further validation assessment of six candidate differentially expressed genes (DEGs)—*Cd74*, *Lpl*, *Ifi44*, *Fzd9*, *Sat1* and *Wwc1*—demonstrated a similar trend at gene transcriptional and protein levels as observed in RNA-sequencing results. Mechanistically, GE treatment-induced *Cd74* downregulation regulated the NF-κB/Bcl-xL/TAp63 signal pathway, which may contribute to soybean GE-mediated therapeutic effects on TNBC tumors. Additionally, our findings revealed that GE can modify expression levels of key epigenetic-associated genes such as DNA methyltransferases (*Dnmt3b*), ten-eleven translocation (*Tet3*) methylcytosine dioxygenases and histone deacetyltransferase (*Hdac2*), and their enzymatic activities as well as genomic DNA methylation and histone methylation (H3K9) levels. Collectively, our investigation shows high significance for potential development of a novel therapeutic approach by using bioactive soybean GE for TNBC patients who have few treatment options.

## 1. Introduction

Breast cancer is the most common malignancy and leading cause of mortality among women worldwide [1]. Triple-negative breast cancer (TNBC) is characterized by a subtype of invasive breast cancer that does not express three important molecular markers including estrogen receptor (ER), progesterone receptor (PR) and human epidermal growth factor receptor 2 (HER-2) [2]. TNBC occurs in about 10–15% of all diagnosed breast cancers and more likely affects premenopausal young women under 40 years old, African Americans, Hispanics, and/or those with a *BRCA1* gene mutation [3,4]. TNBC is highly aggressive as compared to the hormone receptor-positive (luminal A and luminal B) and HER-enriched (HER2+) breast cancer [5]. TNBC tumors have a worse prognosis when associated with a higher rate of early recurrences, often with distant metastases to brain and visceral organs, eventually resulting in a shorter median survival time and death [6]. Due to its molecular etiology, these tumors do not respond to endocrine therapy or molecular target-directed therapies. Therefore, there is an urgent need for optimal therapeutic regimens that can alleviate the risk of this major heath burden by suppressing initiation and progression of TNBC.

The use of bioactive dietary component-based treatment options is gaining focus due to their non-toxic nature and proven anti-cancerous properties [7]. Epidemiological studies have shown that Asian women who consume high amounts of soybean products have a lower risk of breast cancer as compared to Caucasian women with a low intake of soybean products [8]. Genistein (GE) is the most predominant bioactive isoflavone, found mainly in soybean products and other food sources such as lupin, fava beans, kudzu and psoralea. GE acts as a potent chemopreventive and therapeutic agent against various types of cancers including breast, prostate, and lung cancer [9,10]. The mechanisms by which GE elicits its anti-carcinogenic properties involve inhibition of cellular proliferation, angiogenesis, and metastasis as well as induction of apoptosis, differentiation, and cell cycle arrest at the G2-M phase [11,12,13]. Specifically, it is known to inhibit breast carcinogenesis by regulation of several signaling pathways such as downregulation of VEGF, HIF-1α, MMPs, fibronectin, JNK, ERK/PI3K/AKT axis and upregulation of p21 [11,13].

Epigenetics encompasses heritable and reversible changes in gene expression without alterations in the underlying DNA sequence [14], among which DNA methylation and covalent histone modifications play ubiquitous roles in cancer. Recent investigations have implicated the ability of GE to regulate epigenetic mechanisms that are often involved with reversing silenced expression of tumor suppressor genes, leading to cancer prevention/therapeutic effects [15]. Our previous studies have shown that GE significantly inhibited transcription of *hTERT* (human telomerase reverse transcriptase) through regulation of major DNA methyltransferases (DNMT1, 3a and 3b) acting as epigenetic enzymes in control of DNA methylation processes, resulting in inhibition of MCF10AT benign and MCF-7 breast cancer cells [16]. Additionally, dietary GE can enhance the anti-tumor properties of hormone-ablation treatment by re-sensitizing TNBC to tamoxifen treatment through *ERα* reactivation via regulation of epigenetic mechanisms [17]. GE also inhibited breast tumorigenesis by increasing the expression of two key tumor suppressor genes, *p21^WAF1^* and *p16^INK4a^*, in precancerous breast cells and breast cancer cells [18]. Moreover, GE treatment induced alterations of histone modifications in the promoter regions of these genes and repressed tumor growth in established breast cancer xenograft [18].

There has been extensive interest in applying the patient-derived xenograft (PDX) model as an advanced preclinical mouse model to understand the underlying biological mechanisms and treatment approaches for cancer [19]. The PDX model is established by direct transplantation of a tumor slice excised from a cancer patient into an immunodeficient mouse [20]. The growth of tumor in the PDX model resembles similar physiological microenvironmental conditions (O_2_, hormonal and nutritional levels), and maintains principal heterogeneity as the tumor-originated site in a patient [21]. Moreover, the genetic and epigenetic aberrations in the primary tumor from the patient are retained in the implanted tumor in a PDX model [22]. Studies have reported well-established PDX models that have been applied in various cancers including breast, prostate, pancreas, colorectal and lung cancer [23,24,25,26,27]. Therefore, by using a preclinical TNBC PDX mouse model that mimics the process of in situ human TNBC development, our study may help to develop a novel therapeutic strategy for conventionally refractory TNBC patients.

Herein, we investigated the efficacy of bioactive dietary soybean GE treatment in preclinical TNBC PDX mouse models and the underlying impacts on epigenetic mechanisms. We evaluated the therapeutic effects of GE by assessing the tumor weight and tumor volume in immunodeficient female mice bearing two different TNBC PDXs—BCM-3204 and TM00091. We employed RNA-seq analysis to elucidate the potential mechanisms linking GE to its enhanced breast cancer chemotherapeutic potential in TNBC PDX. We further validated the expression of key differentially expressed genes (DEGs) by RT-PCR and Western blot and studied the impact of GE on down-stream target genes as well as the signal pathway of a candidate gene, *Cd74*. We also investigated the effect of GE on various epigenetic-associated key gene expressions and important epigenetic enzymatic activities as well as other pivotal epigenetic processes including global DNA hydroxymethylation and histone methylation. Our results provided important evidence that GE was effective in delaying TNBC development by induction of several tumor-associated genes and regulation of epigenetic modulations, including influencing key epigenetic-related gene expression, DNA methylation and histone methylation processes.

## 2. Materials and Methods

### 2.1. Animals

Two established TNBC PDX models—BCM-3204 (triple-negative tumors from a Caucasian female surgically implanted into NSG mice as described in [28]) kindly provided by Dr. Kai Jiao, and TM00091 (NSG mice engrafted with invasive ductal carcinoma of a 45-year Caucasian female) from Jackson Laboratory—were used in our study. Nonobese diabetic (NOD)/SCID/IL-2γ-receptor null (NSG) (NOD.Cg-*Prkdc^scid^ Il2rg^tm1Wjl^*/SzJ) immunodeficient female mice from the Jackson Laboratory were used to further develop TNBC PDX models. NSG colonies were maintained in a research barrier facility within a 12 h light/dark cycle, 24 ± 2 °C temperatures, and 50 ± 10% humidity. Housing and care of the animals was in accordance with the guidelines established by the University of Alabama at Birmingham Animal Resource Facility.

### 2.2. Animal Diets

We used two diets in the study: control diet (phytoestrogen-free customized AIN-93G diet with 7% corn oil substituted for 7% soybean oil) and GE diet (customized AIN-93G diet supplemented with GE at a concentration of 250 mg/kg) as carried out previously [17,18,29]. The details of the dietary ingredients and nutrition profiles have been provided in File S1 and File S2. Dietary treatment began when primary tumor reached 2 mm in diameter, which represents a therapeutic strategy. Customized GE or corn oil diet in pellets were obtained from Testdiet (St. Louis, MO, USA). Diets were sterilized by irradiation (20–50 kGY), which did not introduce any microorganisms into the experimental animals.

### 2.3. Tumor Implantation, Observation, and Collection

**Figure 1:**
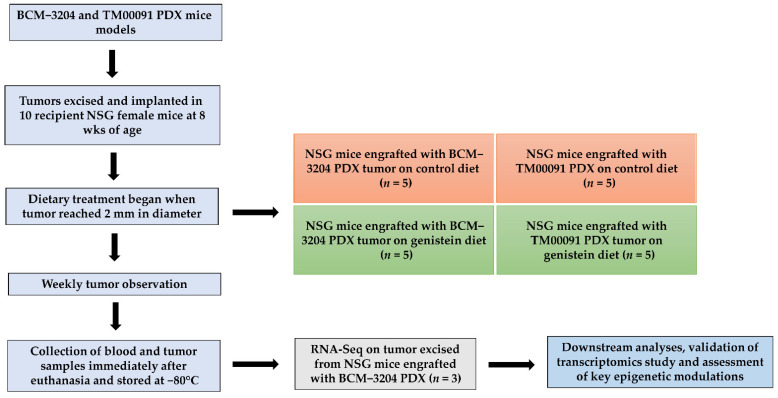
An overview of experimental design of NSG mice bearing BCM−3204 or TM00091 PDX on GE dietary treatment.

The experimental design is illustrated in Figure 1. First, the mammary tumor from live donor of BCM-3204 and TM00091 PDX mice was minced into ~2 mm^3^ tumor tissues. These sliced tumor tissues were orthotopically implanted into mammary fat pads to generate mouse xenografts in 10 recipient NSG female mice at the eighth week of age as reported before [28]. When primary tumor outgrowth reached 2 mm in diameter, mice were randomly divided into two treatment groups (5 mice/group/patient) and administered either control or GE diet ad libitum in the form of pellets. All animals were kept on a regular AIN-93G diet prior to dietary intervention.

The mice were palpated weekly and tumor growth was measured by calipers. Tumor volumes were calculated as tumor volume (cm^3^) = 0.523 × (length (cm) × width^2^ (cm^2^)), as described previously [17,18,30,31]. Animals were sacrificed when all mice from the control group had an average tumor diameter exceeding 1.0 cm. At the endpoint, tumors were excised, weighed and frozen in −80 °C for further analyses. All animal studies were reviewed and approved by the Institutional Animal Use and Care Committee of the University of Alabama at Birmingham (IACUC Animal Project Number: 20671).

### 2.4. RNA Sequencing (RNA-Seq) Analysis

The total RNAs from BCM-3204 PDX were extracted using TRIzol^®^ LS Reagent (Invitrogen, Waltham, MA, USA) according to the manufacturer’s protocol. The concentration of RNA samples was quantified using a NanoDropTM One Spectrophotometer (Thermo Scientific, Wilmington, DE, USA) and integrity was determined with an RNA Nano bioanalyzer chip. Further, randomly selected isolated RNA samples (*n* = 3/group/patient) were analyzed for RNA-seq analysis in UAB Genomics Core Laboratories. The RNA-seq pair-end libraries were sequenced on the Illumina NextSeq 500 platform (Illumina Inc., San Diego, CA, USA). The quality of the raw Fastq was assessed using FastQC (v0.11.4). Subsequently, the obtained RNA-seq fastq reads were aligned to the UCSC Genome Browser human GRCh38/hg38 reference sequence using Kallisto with their default parameter settings. The aligned BAM files were further processed using Kallisto (v 0.43.1-intel-2017a) [32]. Further, an abundance (or transcription) level estimates file was generated for each sample/treatment group, which was used for input in the tximport package [33] of R (v3.6.1).

### 2.5. Differentially Expressed Gene (DEG) Analysis

We utilized R/Bioconductor package Limma (version 3.6.1) to identify transcriptional level changes in PDX tumors (3 mice/group) under control or GE treatment [34]. The significant threshold of |log2(fold-change)| > 2 and false discovery rate (FDR) < 0.01 were set as cutoff for identification of DEGs between the two groups.

### 2.6. Gene Set Function Enrichment

For Gene Ontology (GO) enrichment assessment, we utilized significant transcripts at the transcriptomic level (*p* < 0.05). To further investigate associations of genes with GO terms, functional enrichment was performed using the web-based tool PANTHER (version 10.3) with a significance level of 5% FDR.

### 2.7. Quantitative Real-Time PCR

The complementary DNA (cDNA) was synthesized by reverse transcribing the total RNA with an iScript cDNA Synthesis kit (Bio-Rad, Hercules, CA, USA). Specific gene primers for *Cd74*, *Lpl*, *Ifi44*, *Wwc1*, *Sat1*, *Fzd9*, *TAp63*, *NF-κB*, *Bcl-xL*, *Dnmt1*, *Dnmt3b*, *Hdac2*, *Hdac3, Hdac8, Tet1, Tet2, Tet3* and *Gapdh* were synthesized and purchased from Integrated DNA Technologies (Coralville, IA, USA). The details of primer sequences used are listed in Table S1. All real-time PCR reactions were performed in triplicate on three randomly selected independent samples from each group using SsoAdvanced Universal SYBR Green Supermix (Bio-Rad, Hercules, CA, USA). The *Gapdh* gene was amplified in parallel as an endogenous control. The real-time PCR reaction was analyzed in a BioRad CFX Connect Real-time System where the thermal cycling was initiated for 4 min at 94 °C followed by 35 cycles of PCR (94 °C for 15 s; 60 °C for 30 s, 72 °C for 30 s).

### 2.8. Western Blot Analysis

About 50 mg of frozen TNBC PDX tumors were used to extract total protein with T-PER Tissue Protein Extraction Reagent (Thermo Fisher Scientific, Waltham, MA, USA) according to the manufacturer’s guidelines. The protein concentration was evaluated by Bradford Assay. The equal amount of denatured protein extracts was separated through electrophoresis in 4–15% NuPAGE Tris-HCl precast gels (Invitrogen, Waltham, MA, USA) and transferred onto nitrocellulose membranes. Membranes were then probed with primary antibodies including Cd74, Sat1, TAp63, Bcl-xL, Dnmt1, Dnmt3a, Dnmt3b, Tet2, Tet3, Hdac1, Hdac2, Hdac3, Hdac8 (Cell Signaling Technology, Danvers, MA, USA), Tet1, Ifi44, Fzd9, Wwc1 (Thermo Fisher Scientific, Waltham, MA, USA) and NF-κB and Lpl (Santa Cruz, Dallas, TX, USA). β-actin served as an internal control for each membrane. Immunoreactive bands were visualized using Clarity Max^TM^ Western ECL Blotting Substrates (Bio-Rad, Hercules, CA, USA) and images were captured using ChemiDoc^TM^ Imaging Systems (Bio-Rad, Hercules, CA, USA). The protein expression levels were quantified using Image J software (v1.53e).

### 2.9. DNA Methyltransferase (DNMT) and Histone Deacetylase (HDAC) Activity Assay

Nuclear protein from TNBC PDX tumors was extracted using the EpiQuik Nuclear Extraction Kit (EpiGentek, Farmingdale, NY, USA) according to the manufacturer’s guideline. Nuclear extracts were then used for determination of overall DNMT and HDAC enzymatic activities by using the EpiQuik DNMT Activity/Inhibition Assay Ultra Kit (EpiGentek, Farmingdale, NY, USA) and EpiQuik HDAC Activity/Inhibition Assay Kit (EpiGentek), respectively.

### 2.10. Global DNA Methylation, Hydroxymethylation and Histone Methylation Analysis

DNA extracts of mammary tumor tissues were prepared using DNeasy Blood & Tissue Kits (Qiagen, Germantown, MD, USA) and the manufacturer’s procedure was followed. The NanoDropTM One Spectrophotometer was used to quantify DNA yields and quality. The global DNA methylation status was specifically indicated by the levels of 5-methylcytosine (5-mC) in total DNA and was measured by the MethylFlash Methylated DNA 5-mC Quantification Kit from EpiGentek. In addition, the MethylFlash Hydroxymethylated DNA 5-hmC Quantification Kit (EpiGentek, Farmingdale, NY, USA) was used to quantify global hydroxymethylation status in total DNA samples. Moreover, histone methylation specific to H3K27 and H3K9 residues was evaluated by using EpiQuik Histone Methyltransferase Activity/Inhibition Assay Kits (H3K27) and (H3K9), respectively. The manufacturer’s protocols were followed accordingly.

### 2.11. Statistical Analysis

Power calculation and sample size for the animal study were evaluated by using the Chi-square test, as a sample size of 5 mice/group/patient will give us 95% power for detecting treatment effect at a significance level of 0.05 (α = 0.05) based on our previous GE studies in mice [17,18]. Statistical analyses were performed with SPSS software (version 24.0) (SPSS Inc., Chicago, IL, USA). All experiments were analyzed with a Two-tailed Student’s *t*-test. Results were represented as means ± SE from at least three independent sets of experiments. The results were considered statistically significant when *** *p* < 0.001, ** *p* < 0.01 and * *p* < 0.05.

## 3. Results

### 3.1. Dietary GE Inhibited TNBC Growth in PDX Models

The NSG mice are characterized by a lack of B cells, T cells, and functional natural killer cells. This severe immunodeficiency makes these mice an ideal PDX recipient for engraftment with malignant TNBC tissue [28,35]. The previous studies of our lab [16,17,18] have indicated safe and efficacious administration of GE against breast cancer in other strains of mice. To further determine the effects of the GE diet on general wellness, body weight, and physiological changes such as general behavior, eating behavior, grooming behavior and social interaction of NSG mice were recorded weekly. Our results showed that there were no adverse effects on the body weight (Figure S1) and other general wellbeing parameters, suggesting the soybean GE is safe to use in NSG mice.

**Figure 2:**
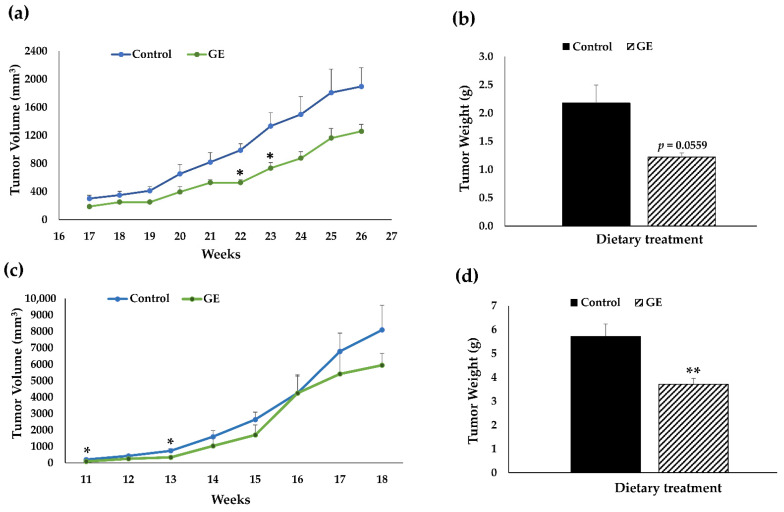
Tumor inhibitory effects of dietary GE on tumor growth in TNBC BCM−3204 PDX mouse model and TM00091 PDX model. Female NSG mice (5 per group) were implanted PDX tumors at 8 weeks of age and administrated either control or GE diet when primary tumor outgrowth reached 2 mm in diameter. The relevant treatments continued until termination of the experiment. Tumor volumes and weights were observed weekly in BCM−3204 PDXs (**a**,**b**) or TM00091 PDXs (**c**,**d**). (**a**,**c**) depict tumor volume and (**b**,**d**) depict tumor weight at termination. (* *p* < 0.05, ** *p* < 0.01). Data are presented as mean ± standard error (SE). GE, genistein.

From tumor growth observation, we found that GE treatment was effective in reducing tumor volume, especially at 22 wks and 23 wks of age, in NSG mice bearing BCM-3204 PDX as compared to the control diet (Figure 2a). From tumor weight, we found a notable decrease (*p* = 0.0559) with administration of dietary GE in BCM-3204 PDX as depicted in Figure 2b. GE treatment can also significantly decrease tumor volume at 11 wks and 13 wks in NSG mice engrafted with TM00091 PDX (Figure 2c). Furthermore, a significant decrease in tumor weight (Figure 2d) was induced by GE treatment in TM00091 PDX model. Overall, our results indicate that GE was effective in delaying tumor development in both PDX models from different TNBC patients, as demonstrated by its ubiquitous anti-cancer effects in reducing tumor volume and tumor weight in comparison to the control group without any negative impact on general health. Because the impact of GE on suppressing tumor growth was more promising in BCM-3204 than the TM00091 PDX model, we therefore conducted the subsequent transcriptomics analyses in the BCM-3204 PDX model.

### 3.2. Genome-Wide Transcriptomic Alterations Induced by GE Administration in BCM-3204 PDX Model

**Figure 3:**
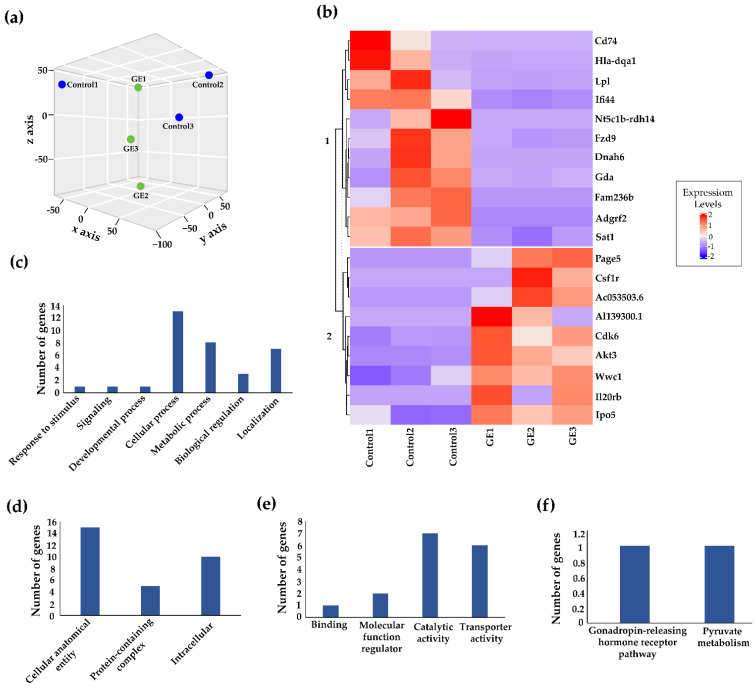
Transcriptome analyses and distribution of GO slim terms across dietary treatment groups. (**a**) 3-D PCoA plot demonstrates spatial arrangements of each sample from control and GE treatment groups. (**b**) Heatmap represents nine upregulated and eleven downregulated genes induced by dietary GE based on *p* value. Each row corresponds to differentially expressed transcripts and each column represents biological replicates in control (*n* = 3) and GE (*n* = 3) treatment group. Purple color denotes lower expression levels and red color denotes higher expression levels. Bar plot distribution of GO slim terms of differentially expressed transcripts related to the dietary GE treatment in (**c**) biological process, (**d**) cellular components, (**e**) molecular functions and (**f**) pathways. The column of bar plot represents the total number of differentially expressed genes.

To further discover the underlying mechanisms, we performed comprehensive genome-wide transcriptomic analysis to identify key genes that expression changes may link soybean GE to its therapeutic effects against TNBC. We used PDX tumors from NSG mice engrafted with BCM-3204 and performed RNA-seq analysis as carried out previously [36,37]. The obtained RNA-seq data were transformed for linear modeling and a boxplot was generated to identify the samples’ outliers across different treatment groups (Figure S2a). As a result, no outliers were found, and all the samples were included in further analysis. The normal distribution of samples was confirmed by plotting a histogram (Figure S2b). Subsequently, a three-dimensional Principal Coordinates Analysis (PCoA) plot was generated based on the gene expression profile of each sample among both control and dietary GE groups (Figure 3a). Based on the spatial arrangements in the PCoA plot, no significant overlap between transcripts was observed, implying a shift in gene expression profile with the administration of GE as compared to the control group.

Our RNA-seq analysis discovered a total of 14,941 transcripts (genes) (File S3) in response to dietary GE treatment, among which 20 transcripts showed the most significantly differential expression. We generated a heatmap of the significantly upregulated and downregulated genes with rows corresponding to DEGs and columns corresponding to biological replicates in control and GE treatments (Figure 3b). Among a total of 20 significant differentially expressed genes, 9 (45%) genes were upregulated and 11 (55%) were downregulated using the cutoff criteria of 5% FDR and fold-change >2. Detailed information on the top 20 DEGs induced by dietary GE treatment is outlined in Table 1.

We used PANTHER software to conduct GO SLIM subset analyses, which can reveal major clusters within various biological processes, cellular components and molecular functions. Among the biological process classification, we found “cellular processes” and “metabolic processes” were the most frequent terms (Figure 3c). Similarly, the major subsets in the cellular component category were “cellular anatomical entity” and “intracellular” (Figure 3d). In the molecular function category, the most abundant terms were related to “catalytic activity” and “transporter activity” (Figure 3e). Additionally, we found “gonadotropin-releasing” and “pyruvate metabolism” pathways were associated with these key DEGs (Figure 3f). Thus, soybean GE appears to impact these important signal pathways, which may contribute to its therapeutic effects against TNBC progression.

### 3.3. Validation Analyses of Target Gene Expression at Transcriptional and Translational Levels

Among the most significantly DEGs in Table 1, with transcriptomics analyses, we chose six target genes including *Cd74*, *Lpl*, *Ifi44*, *Fzd9*, *Sat1* and *Wwc1* based on their role in cancer and epigenetic mechanisms for further validation studies (Table 2).

**Figure 4:**
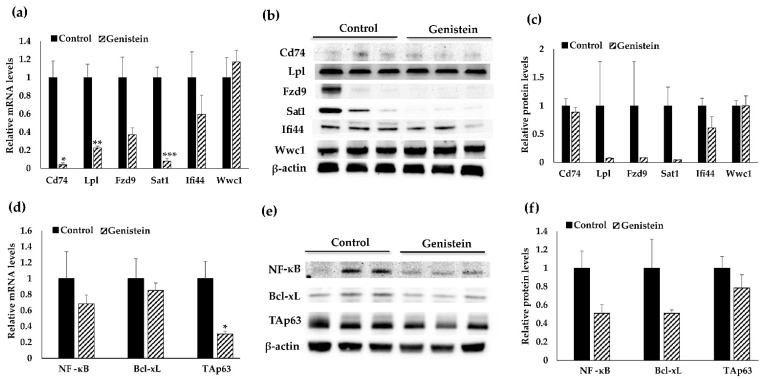
Validation analyses of 6 candidate genes expression and Cd74-regulated signal pathway. Real-time PCR and Western blot analysis were performed in triplicates to evaluate gene expression changes of Cd74, Lpl, Fzd9, Sat1, Ifi44, Wwc1, NF-κB, TAp63 and Bcl-xL in TNBC PDX tumors of BCM-32045 from both control and GE treatment groups (*n* = 3/patient/group). (**a**) Relative gene expression in transcriptional level in 6 identified target genes. (**b**) Protein expression of 6 identified target genes, (**c**) Quantification of the target protein levels. (**d**–**f**) Cd74-regulated signal pathway genes including NF-κB, Bcl-xL and TAp63 in transcriptional (**d**) or protein (**e**) levels as well as protein quantification of these genes (**f**). The transcription or protein expression levels were normalized to the relevant housekeeping control and calibrated to control group. Representative blots were selected from experiments that were repeated three times. Columns, mean; bars, SE; * *p* < 0.05, ** *p* < 0.01, *** *p* < 0.001 significantly different from the control group.

To validate the results of our genome-wide transcriptomic analysis, we evaluated transcriptional and protein levels of six identified key genes by using real-time quantitative RT-PCR and Western blotting. We found that the mRNA and protein expression patterns in these target genes were consistent with the RNA-seq results (Figure 4a–c). For instance, GE treatment resulted in significant downregulation of *Cd74*, *Lpl* and *Sat1* at the transcriptional level. The treatment also led to downregulation of *Fzd9* and *Ifi44* at the transcriptional level (Figure 4a). We further assessed the gene changes in protein levels and observed decreased expression in protein levels of Cd74, Lpl, Fzd9 Sat1 and Ifi44 (Figure 4b,c). Although protein expression change was not statistically significant, the trends were obviously consistent with what have been seen in transcriptional levels.

### 3.4. Cd74-Regulated Signaling Pathway May Contribute to GE Diet-Induced Therapeutic Effects against TNBC

We further investigated the potential mechanism underlying the anti-cancerous effects of GE on TNBC by exploring the Cd74-regulated signal pathway. Cd74 was identified as a candidate DEG through RNA-seq analysis and has been found to be frequently overexpressed in multiple cancers. Mechanistically, Cd74 binds to MIF, a multifunctional cytokine that expresses on multiple cells, resulting in the induction of downstream signaling cascades including the induction of intramembrane cleavage, activation of p65 member in the nuclear factor kappa B (NF-κB) family and elevation of the p53-related tumor protein p63 (TAp63) [49,50,51]. Furthermore, NF-κB is a key proinflammatory transcription factor that is known to be involved in the pathogenesis of breast cancer [52]. We therefore evaluated the expression of NF-κB by using quantitative RT-PCR and Western blot. As illustrated in Figure 4d–f, the administration of GE resulted in decreased NF-κB expression at both transcriptional and translational levels (*p* = 0.090) in TNBC PDXs.

The Cd74/NF-κB axis triggers Bcl-xL, which is a member of the Bcl-2 gene family and acts as an anti-apoptotic factor [53]. Investigations have reported for the upregulation of Bcl-xL in several cancers including hepatocellular cancer [54], non-small cell lung cancer [55] and breast cancer [56]. Importantly, our assessment revealed decreased Bcl-xL expression at the gene and protein levels (Figure 4d–f) in GE-fed PDX mice.

TAp63 is an isoform of p63 that plays a key role in mammary gland development and homeostasis [57]. It has been reported that activation of Cd74 by MIF can result in upregulation of TAp63, which further leads to increased expression of integrin VLA-4, resulting in enhanced migration and survival of chronic lymphocytic leukemia cells [51]. Our results showed that mRNA and protein levels of *Tap63* were downregulated (Figure 4d–f) by the treatment of GE as compared to the control. These findings demonstrated that GE treatment may mediate Cd74 and its downstream target gene expressions, which may contribute to its anti-tumor effects on TNBC.

### 3.5. Dietary GE Treatment Resulted in Expression Changes in Multiple Epigenetic-Related Genes

**Figure 5:**
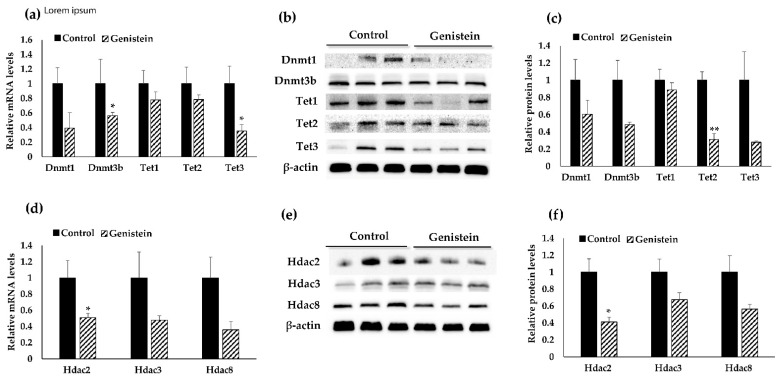
Assessment of transcriptional and protein levels of key epigenetic-related genes. (**a**,**d**) Dnmt1, Dnmt3b, Tet1, Tet2, Tet3, Hdac2, Hdac3 and Hdac8 gene transcriptional levels by real-time RT-PCR in TNBC PDX tumors in control and GE treatment groups, (**b**,**e**) Protein levels of these key epigenetic-related genes by Western blot, (**c**,**f**) Histograms showed quantification of protein levels. Gapdh or β-Actin was used as internal control. Data shown are representative of three independent experiments (*n* = 3/patient/group). Columns, mean; Bars, SD; * *p* < 0.05, ** *p* < 0.01, significantly different from control group.

In an effort to understand GE-mediated epigenetic mechanisms underlying our observations, we assessed gene expressions and protein levels of crucial epigenetic modifiers including Hdacs and Dnmts in GE-treated BCM-3204 PDX tumors. Initially, we focused on Dnmts as GE is a known epigenetic modifier specifically acting as a potent inhibitor for Dnmt1, Dnmt3a and Dnmt3b [16,58]. Our results showed that GE treatment significantly decreased *Dnmt3b* mRNA expression (Figure 5a) and the change was nearly significant (*p* = 0.091) at the Dnmt3b protein level (Figure 5b,c). This finding was consistent with our previous studies indicating anti-cancerous effects of GE on the regulation of DNA methylation [16,17].

We further assessed expression of ten-eleven translocation (Tets) methylcytosine dioxygenase enzymes that play an integral role in the DNA demethylation process. We also evaluated gene expression of Hdacs including Hdac2, Hdac 3 and Hdac 8 that participate in histone deacetylation processes. Our finding revealed a significant decrease in *Tet3* mRNA expression (Figure 5a) and Tet2 protein expression (Figure 5b,c). Further investigation showed a significant decrease in *Hdac2* at both the transcriptional and translational levels (Figure 5d–f). We also found a nearly significant change in Tet3 (*p* = 0.096) and Hdac3 (0.097) protein levels. These results collectively suggest important roles of the GE diet in inhibition of Dnmts, Tets and Hdacs in triple-negative breast carcinogenesis.

### 3.6. GE Influenced Global Epigenetic Profiles

**Figure 6:**
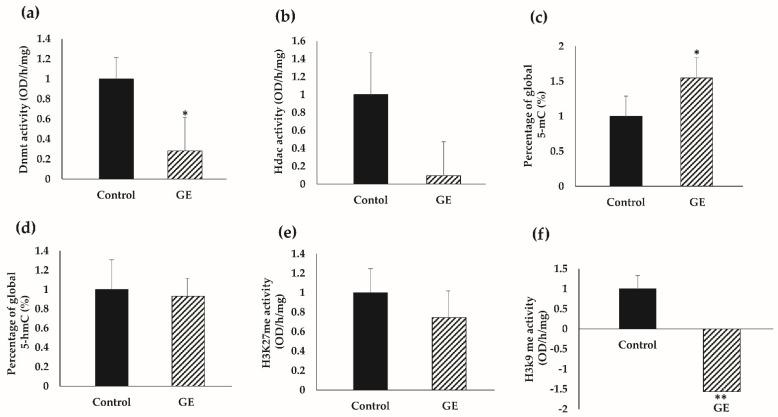
Global epigenetic profiles in response to GE treatment. (**a**) Dnmt activity. (**b**) Hdac activity. (**c**) Global DNA methylation levels as represented global 5-mC percentage in genomic DNA. (**d**) Global DNA hydroxymethylation levels as represented genomic 5-hmC content in total DNA. (**e**) Histone methyltransferase (H3K27) activity. (**f**) Histone methyltransferase (H3K9) activity. Results were in triplicate from three randomly selected mouse TNBC tumors from each treatment group. Columns, mean; bars, SE; ** *p* < 0.001, * *p* < 0.05, significantly different from the control group. GE, genistein.

We further sought to determine epigenetic-driven mechanistic insights with consumption of dietary GE on TNBC therapy. In Figure 6a, we demonstrated a significant decrease in enzymatic activity of Dnmts, verifying our findings that the GE diet may have strong influence on DNA methylation in TNBC tumors. Further, we observed a decrease in Hdac enzymatic activity; however, this change was not significant (Figure 6b).

We further investigated global DNA methylation by detecting 5-methylcytosine (5-mC) content and DNA hydroxymethylation (5-hmC) status in the genomic DNA isolated from TNBC tumors. Paradoxically with DNMTs activity but consistently with decreased Tet gene expression pattern, exposure to dietary soyabean GE led to a significant increase in global 5-mC percentage in the genomic DNA (Figure 6c). However, we observed no effect on global 5-hmC percentage in the genomic DNA of GE-treated TNBC tumors (Figure 6d).

Post-translational histone covalent modifications such as methylation of histone residues play a profound role in cancer initiation and progression. We specifically focused on two histone methylation markers, histone H3 at lysine 27 represented by H3K27me and histone H3 at lysine 9 indicated by H3K9me, respectively. Our observation indicated a significant decrease in histone methylation at H3K9 residue in GE-treated PDX mice as compared to the control group (Figure 6f). Further, we observed a decrease in H3K27me status; however, this change was not significant. Taken together, our findings suggest that administration of GE may impact important epigenetic enzymatic activities, resulting in global epigenetic profile changes, which may contribute to GE-induced breast tumor inhibitory effects in TNBC PDXs.

## 4. Discussion

TNBC is one of the most aggressive breast cancer subtypes with few treatment options. There is an urgent need for exploration of novel therapeutic strategies for TNBC treatment. Interest in phytopharmaceuticals is growing rapidly as numerous investigations have demonstrated that nutritional factors play fundamental roles in preventive and therapeutic effects against various types of cancers. Genistein (GE) is a major isoflavone found in soybean products including soy milk, soy protein and tofu, and acts as a potent chemopreventive agent against breast cancer. Specifically, GE treatment induces apoptosis and cell-cycle arrest and targets various signaling pathways including Akt, HIF-1α and VEGF pathways. GE has shown synergistic effects when combining with other conventional anticancer drugs in breast cancer treatment [13,59,60]. Pintova et al. tested the safety of GE when co-administrated with Fluorouracil and Oxaliplatin (FOLFOX) with or without the anti-angiogenic agent, Bevacizumab, in colorectal cancer patients. They observed that combinations of GE with FOLFOX or FOLFOX–Bevacizumab were safe and tolerable. Furthermore, their findings suggested that combination of GE with conventional chemotherapy may have a profound impact on treatment of metastatic colorectal cancer as compared to standard chemotherapy [61].

More importantly, GE participates in epigenetic regulation through, at least in part, acting as an inhibitor of Dnmts or Hdacs [16,58]. In the present study, we investigated the efficacy of dietary GE treatment in TNBC and explored the potential mechanisms by using a novel PDX mouse model, which represents a superior preclinical model system for drug testing [62]. Our results showed that soybean GE significantly inhibited TNBC development in both tested PDX models as compared to the control group without showing any harmful effect. With RNA-seq analysis, we found GE treatment induced transcriptomic profile changes in NSG mice engrafted with BCM-3204 PDX tumors. We further identified several important DEGs that may influence multiple critical biological processes, cellular components, and metabolic pathways due to GE dietary treatment.

Subsequently, we focused on a group of six candidate genes (*Cd74*, *Lpl*, *Ifi44*, *Sat1*, *Fzd9* and *Wwc1*) that have been reported to be frequently regulated by epigenetic mechanisms and participate in the regulation of important signal pathways during cancer development and progression. *Cd74* is found overexpressed in several types of cancers [63,64,65], suggesting that *Cd74* may serve as a tumor progression marker. *Lpl* is a fatty acid metabolism gene. It is found upregulated in human mammary epithelial cells expressing oncogenic levels of Myc, which is further associated with poor survival rate in TNBC [66]. *Ifi44* is abnormally expressed in head and neck squamous cell carcinoma as compared to normal tissues [46]. Additionally, *Sat1* is found to ameliorate resistance to ionizing radiation and upregulated in brain tumors [45]. *Fzd9* is aberrantly expressed in malignant astrocytoma and gastric cancer [42,43]. The *Wwc1* gene encodes protein Wwc1 (or KIBRA) that has been reported to suppress tumor growth and metastatic potential in TNBC [67]. Validation analyses of these key tumor-related genes at transcriptional and protein levels revealed a similar trend as found in transcriptomic analysis. For example, we observed a significant downregulation of oncogenes such as *Cd74*, *Lpl* and *Sat1* at the transcriptional level and a further decrease in protein levels of Cd74, Lpl, Fzd9, Sat1 and Ifi44 in PDX tumors exposed to the GE diet.

We further sought to understand the role of Cd74 and its regulated signal pathway during the process of TNBC progression. We evaluated the Cd74-regulated signal pathway in the NF-ĸB/Bcl-xL/TAp63 axis. Previous studies have shown that Cd74 upregulates the expression of NF-ĸB, which in turn activates TAp63 and Bcl-xL proteins, thereby enhancing the cancerous cells’ proliferation and differentiation [53,68]. Our results revealed dietary GE-induced downregulation of Cd74 may lead to a downstream gene expression cascade in *NF-ĸB*, *Bcl-xL* and *TAp63* mRNA and protein levels that may contribute to GE-induced therapeutic efficacy in TNBC.

Our study is innovative with respect to the use of two novel PDX mouse models to investigate the treatment efficacy of dietary GE in inhibiting TNBC and identify key tumor-related genes that may be involved in GE-mediated epigenetic machinery. GE and its derived soybean products have been well documented due to the anti-tumor effects through regulation of epigenetic processes and subsequent gene transcriptional profile changes during tumorigenesis. Our assessment on important epigenetic modulators showed a significant decrease in *Dnmt3b*, *Tet3* and *Hdac2* expression, suggesting GE may alter crucial epigenetic modifiers leading to TNBC suppression. Subsequently, we assessed whether GE-induced downregulation of Dnmt3b had an impact on Dnmt enzymatic activity and global DNA methylation status. As expected, GE treatment significantly decreased the Dnmt enzyme activity. However, GE increased the percentage of genomic 5-mC in TNBC PDX-tumors, which may relate to its inhibitory effects on Tets resulting in suppression of DNA demethylation processes. The hypomethylation of genomic DNA has been linked with cancer hallmarks such as genomic instability, aberrant transcription, cell transformation and transposable elements reactivation [69]. Therefore, GE-induced repression of H3K9 methylation may correlate with gene transcriptional activation status. These global epigenomic landmark changes may have a profound impact on regulation of key gene expression profiles that attribute to soybean dietary GE-induced therapeutic effects on TNBC suppression.

Moving forward, we focused on histone methylation, which is catalyzed by histone methyltransferase enzymes involving the addition of methyl groups primarily on the lysine residues. We specifically assessed the levels of histone methylation on H3K27 and H3K9 residues. Our observation indicated a significant decrease in H3K9me and a minor decrease in H3K27me status; however, the latter was not significant. These histone methylation signatures are found to be associated with transcriptional silencing that may aid in breast cancer progression [70]. Therefore, GE-induced repression of H3K9 methylation along with other epigenetic component changes may have a profound impact on TNBC suppression.

Our study outlines the importance of a nontoxic and convenient regimen using dietary GE to suppress TNBC tumors derived from breast cancer patients that could revolutionize breast cancer therapy for hundreds of thousands of women worldwide. Previous epidemiological studies have shown an inverse association between soy intake and risk of breast cancer development [8,71]. Results from clinical studies reported no changes in breast density and mammary epithelium proliferation with the consumption of high-dose GE supplements in breast cancer patients, high-risk women, and healthy women [72,73]. For instance, a one-year randomized, placebo-controlled clinical trial was conducted to assess the effects of GE on cytogenetic biomarkers, showing the protective role of GE in BC development in postmenopausal women [74]. Furthermore, Liu et al. elaborated the importance of individual factors such as menopausal status, estrogen receptor expression pattern, and gene mutations in the patient for consideration of personalized responses to dietary soybean GE intervention [75].

The dietary concentration used in our study is equivalent to 3 cups of boiled soybean per day [76]. Therefore, the concentration of GE diet used in this study is safe, efficacious, and physiologically relevant and has high translational potential. Thus, our study provides an important preclinical foundation to facilitate future clinical trials to use GE in TNBC patients. Overall, our study suggests a therapeutic potential of bioactive soybean GE in refractory TNBC through investigation of potential mechanisms via mediating molecular targets and epigenetic mechanisms. Our study may lead to a novel therapeutic approach by incorporating nutritional intervention and conventional therapy in intractable TNBC. Future studies are warranted to explore further epigenetic mechanisms such as DNA methylomic changes to elucidate potential correlation between gene transcription and DNA methylation in more TNBC patient tumor samples. Moreover, clinical trials will be important to investigate the efficacy and safety of dietary soyabean GE in prevention and treatment of TNBC patients.

## Figures and Tables

**Table 1:** The top DEGs in response to dietary soybean GE treatment assorted by *p* value.

Gene Symbol	Gene Expression Fold Change (log_2_FC)	Average Differential Expression	*p*-Value for Differential Expression	False Discovery Rate (FDR)
*Cd74*	−4.760	2.879	2.20 × 10^−7^	0.003
*Lpl*	−2.806	2.294	2.26 × 10^−6^	0.017
*Ifi44*	−2.191	2.100	8.23 × 10^−6^	0.027
*Dnah6*	−6.634	−3.412	5.63 × 10^−6^	0.027
*Il20rb*	6.115	−3.578	9.12 × 10^−6^	0.027
*Wwc1*	0.903	5.994	1.27 × 10^−5^	0.028
*Sat1*	−0.823	6.315	1.49 × 10^−5^	0.028
*Al139300.1*	5.984	−3.559	1.32 × 10^−5^	0.028
*Akt3*	1.335	5.583	2.34 × 10^−5^	0.037
*Ipo5*	0.935	7.051	2.46 × 10^−5^	0.037
*Gda*	−1.759	2.098	3.27 × 10^−5^	0.039
*Fzd9*	−1.186	4.141	4.89 × 10^−5^	0.039
*Cdk6*	0.903	9.485	4.07 × 10^−5^	0.039
*Hla-dqa1*	−5.147	−1.468	4.60 × 10^−5^	0.039
*Adgrf2*	−4.405	−3.498	3.85 × 10^−5^	0.039
*Page5*	4.595	−3.685	3.63 × 10^−5^	0.039
*Fam236b*	−4.509	−3.722	4.96 × 10^−5^	0.039
*Ac053503.6*	4.624	−3.537	5.12 × 10^−5^	0.039
*Csf1r*	5.257	−3.778	3.26 × 10^−5^	0.039
*Nt5c1b-rdh14*	−4.914	−3.895	5.25 × 10^−5^	0.039

**Table 2:** Identified key genes showed significantly differential expression in response to GE treatment, and their role in epigenetic mechanisms and cancer.

Gene	Function in Cancer	Epigenetic Regulation	RNA-Seq
Cluster of Differentiation 74 (*Cd74*)	Cd74 is overexpressed in breast cancer patients. It is also found to be significantly correlated with lymph node metastasis in TNBC [38]	Epigenetic mechanisms play a role in Cd74 expression via *Cd74* promoter methylation [39]	DEG, significant decrease (4.76 fold)
Lipoprotein lipase (*Lpl*)	Breast cancer and sarcoma cells express and secrete active Lpl enzyme to acquire fatty acids from the blood circulation, which facilitate growth of these cells [40]	Epigenetic changes at the promoter regions may alter expression of the *Lpl* gene and may play an important role in prostate cancer development [41]	DEG, significant decrease (2.8 fold)
Frizzled 9 (*Fzd9*)	Oncogene. Overexpressed *Fzd9* is found in various types of cancer [42,43]	Hypermethylated *Fzd9* is associated with hormone receptor positive, luminal A, or p53 wild-type breast cancers [44]	DEG, significant decrease (1.18 fold)
Spermidine/spermine-N1-acetyltransferase 1 (*Sat1*)	Sat1 overexpression is correlated with poor clinical outcomes [45]	Sat1 is involved in acetylation of histone H3, resulting in chromatin remodeling and regulation of gene expression [45]	DEG, significant decrease (0.82 fold)
Interferon-Induced Protein 44 (*Ifi44*)	Oncogene. Overexpressed *Ifi44* is found in head and neck squamous cell carcinoma and functioned heterogeneously in tumor formation and progression [46]	*Iifi44* promoter hypomethylation can distinguish systemic lupus erythematosus patients from healthy persons, promising to be first novel epigenetic diagnostic marker [47]	DEG, significant decrease (2.19 fold)
WW and C2 domain containing 1 (*Wwc1*)	Tumor-suppressor gene. Low *Wwc1* expression is associated with aggressive breast cancer and poor survival outcomes [48]	DNA methylation is negatively correlated with *Wwc1* expression [48]	DEG, significant increase (0.90 fold)

## Data Availability

The data supporting reported results can be found at http://www.ncbi.nlm.nih.gov/bioproject (accessed on 23 September 2021) with submission ID SUB10415300 and Bioproject ID PRJNA765810.

## Supplementary Materials

The following are available online at https://www.mdpi.com/article/10.3390/nu13113944/s1, File S1: Detailed dietary ingredients and nutrition composition of customized control diet, File S2: Detailed dietary ingredients and nutrition profiles of customized GE diet, Table S1: Primer sequences for real-time PCR analysis, Figure S1: Body weight of (a) BCM-3204 PDX, and (b) TM00091 PDX when exposed to GE. Mouse body weight was recorded weekly from 10 wks, and 11 wks in NSG mice bearing BCM-3204 and TM00091 PDX, respectively. Values are means ± SE, *n* = 5/patient/dietary group, Figure S2: (a) This Boxplot shows no outliers and therefore all samples (*n* = 3/group) from dietary treatments were used in the transcriptomics analyses of BCM-3204 PDX tumors. (b) Histogram illustrates normal distribution of total feature by counts among control and GE samples wherein *x*-axis represents the total frequency across 6 samples and *y*-axis represents sample counts, File S3: A list of transcripts in response to GE treatment (*p* < 0.05) and calculated fold change at transcriptome level.

## Abbreviations

Cd74	Cluster of Differentiation 74
cDNA	Complementary DNA
DEGs	Differentially expressed genes
Dnmts	DNA methytransferases
ER	Estrogen receptor
FastQC	FastQ quality control
FC	Fold change
FDR	False-discovery rate
Fzd9	Frizzled 9
GE	Genistein
GO	Gene Ontology
Hdac	Histone deacetylase
HER-2	Human epidermal growth factor receptor 2
hTERT	Human telomerase reverse transcriptase
Ifi44	Interferon-Induced Protein 44
Lpl	Lipoprotein lipase
NF-κB	Nuclear factor kappa B
NOD	Nonobese diabetic
NSG	NOD/SCID/IL-2γ-receptor null
PCoA	Principal Coordinates Analysis
PDX	Patient-derived xenograft
PR	Progesterone receptor
RNA-seq	RNA sequencing
Sat1	Spermidine/spermine-N1-acetyltransferase 1
TAp63	Tumor protein p63
Tet	Ten-eleven translocation
TNBC	Triple negative breast cancer
Wwc1	WW and C2 domain containing 1
