# Supplementary material for: Therapeutic Effects of Dietary Soybean Genistein on Triple-Negative Breast Cancer via Regulation of Epigenetic Mechanisms
Source: Nutrients. 2021 Nov 4;13(11):3944. doi: 10.3390/nu13113944 (PMC8623013; doi:10.3390/nu13113944)
Supplement: Supplementary file 1 [file nutrients-13-03944-s001.zip › File S1.pdf]

AIN-93G w/ 7.0% Corn Oil — 5B9M

DESCRIPTION

Modification of TestDiet® AIN-93G Semi-Purified Diet 57W5 with Corn Oil Replacing Soy Oil at 7.0% of the Diet.

Storage conditions are particularly critical to TestDiet® products, due to the absence of antioxidants or preservative agents. To provide maximum protection against possible changes during storage, store in a dry, cool location. Storage under refrigeration (2° C) is recommended. Maximum shelf life is six months. (If long term studies are involved, storing the diet at -20˚ C or colder may prolong shelf life.) Be certain to keep in air tight containers.

*Product Forms Available**	*Catalog #*
1/2" Pellet	1811961

*Other Forms Available On Re

INGREDIENTS (%)

Corn Starch	39.7486
Casein - Vitamin Free	20.0000
Maltodextrin	13.2000
Sucrose	10.0000
Corn Oil	7.0000
Powdered Cellulose	5.0000
AIN 93G Mineral Mix	3.5000
AIN 93 Vitamin Mix	1.0000
L-Cystine	0.3000
Choline Bitartrate	0.2500
t-Butylhydroquinone	0.0014

FEEDING DIRECTIONS

Feed ad libitum. Plenty of fresh, clean water should be available at all times.

CAUTION:
Perishable - store properly upon receipt.
For laboratory animal use only; NOT for human consumption.

5/29/2009

NUTRITIONAL PROFILE [1]

Protein, %	*18.3*
Arginine, %	0.70
Histidine, %	0.52
Isoleucine, %	0.96
Leucine, %	1.73
Lysine, %	1.45
Methionine, %	0.52
Cystine, %	0.37
Phenylalanine, %	0.96
Tyrosine, %	1.01
Threonine, %	0.77
Tryptophan, %	0.22
Valine, %	1.14
Alanine, %	0.55
Aspartic Acid, %	1.29
Glutamic Acid, %	4.08
Glycine, %	0.39
Proline, %	2.36
Serine, %	1.10
Taurine, %	0.00

Fat, %	*7.1*
Cholesterol, ppm	0
Linoleic Acid, %	4.00
Linolenic Acid, %	0.06
Arachidonic Acid, %	0.00
Omega-3 Fatty Acids, %	0.06
Total Saturated Fatty A	0.89
Total Monounsaturated Fatty Acids, %	1.69
Polyunsaturated Fatty Acids, %	4.06

Fiber (max), % **5.0**

Carbohydrates, % **63.2**

Energy (kcal/g) [2] **3.89**

From:	kcal	%
Protein	0.731	18.8
Fat (ether extract)	0.637	16.4
Carbohydrates	2.528	64.9

Minerals	
Calcium, %	0.51
Phosphorus, %	0.32
Phosphorus (available), %	0.16
Potassium, %	0.36
Magnesium, %	0.05
Sodium, %	0.13
Chloride, %	0.22
Fluorine, ppm	1.0
Iron, ppm	36
Zinc, ppm	35
Manganese, ppm	11
Copper, ppm	6.0
Cobalt, ppm	0.0
Iodine, ppm	0.21
Chromium, ppm	1.0
Molybdenum, ppm	0.14
Selenium, ppm	0.24

Vitamins	
Vitamin A, IU/g	4.0
Vitamin D-3 (added), IU/g	1.0
Vitamin E, IU/kg	75.0
Vitamin K (as menadione), ppm	0.29
Thiamin Hydrochloride, ppm	6.1
Riboflavin, ppm	6.7
Niacin, ppm	30
Pantothenic Acid, ppm	16
Folic Acid, ppm	2.1
Pyridoxine, ppm	5.8
Biotin, ppm	0.2
Vitamin B-12, mcg/kg	29
Choline Chloride, ppm	1,250
Ascorbic Acid, ppm	0.0

1. Based on the latest ingredient analysis information. Since nutrient composition of natural ingredients varies, analysis will differ accordingly. Nutrients expressed as percent of ration on an As-Fed basis except where otherwise indicated.
2. Energy (kcal/gm) - Sum of decimal fractions of protein, fat and carbohydrate x 4,9,4 kcal/gm respectively.

ISO 9001:2000 CERTIFIED

TestDiet
www.testdiet.com
