# Supplementary material for: Therapeutic Effects of Dietary Soybean Genistein on Triple-Negative Breast Cancer via Regulation of Epigenetic Mechanisms
Source: Nutrients. 2021 Nov 4;13(11):3944. doi: 10.3390/nu13113944 (PMC8623013; doi:10.3390/nu13113944)
Supplement: Supplementary file 1 [file nutrients-13-03944-s001.zip › Table S1.pdf]

Table S1. Primer sequences for real-time PCR analysis

Gene	Forward Primer Sequence (5'-3')	Reverse Primer Sequence (5'-3')
Cd74	CTGAGACACCTTAAGAACACCA	TGGCACTTGGTCAGTACTTTC
Lpl	AGTCAGAGCCAAAAGAAGCAG	TTGGTATGGGTTTCACTCTCAG
Ifi44	GCGAAGATTCACTGGATGAAAG	TCGTATTTGTTGAACCAGGGA
Wwc1	CTACATAGACCACACGAACCG	GTCATATGCCTCTTCCCATCC
Sat1	CAGAAGTGCCGAAAGAGCA	CTCATCACGAAGAAGTCCTCAA
Fzd9	GTCTTCCCATCCCTACACG	CGCCAGAAGTCCATGTTGA
TAp63	CAAGAAACGAAGATCCCCAGA	GAGGAAGGTACTGCATGAGTTC
Nf-κB	CGAGCTTGTAGGAAAGGACTG	TGACTGATAGCCTGCTCCAG
Bcl-xL	GCCACTTACCTGAATGACCAC	GCATTGTTCCCATAGAGTTCCA
Dnmt1	GGATGACAGAGAAGACAAGGAG	GCTTTACATTTCCCACACTCAG
Dnmt3B	AAACCCAACAACACGCAAC	TTCTCGGCTCTGATCTTCATC
Hdac2	CATGGCGTACAGTCAAGGAG	CATGCGGATTCTATGAGGCTT
Hdac3	AGACCTCCTGACCTATGACA	TCTCCACATCGCTTTCCTTG
Hdac8	GTCTTAAGTACATCCTTCAATGGC	GTCAAGTATGTCCAGCATCGAG
Tet1	CAAGCGGAAGAATAACTCAACAAC	CGGTTTCACTTTTTACTTCAGGT
Tet2	CATGTTTGGACTTCTGTGCTC	ATTCTCGATTGTCTTCTCTAGTGAG
Tet3	TGCGTCGAACAAATAGTGGA	GTGTAGATGACCTTCTCGATCC
Gapdh	ACATCGCTCAGACACCATG	TGTAGTTGAGGTCAATGAAGGG
