# Supplementary figures and images for: Therapeutic Effects of Dietary Soybean Genistein on Triple-Negative Breast Cancer via Regulation of Epigenetic Mechanisms
Source: Nutrients. 2021 Nov 4;13(11):3944. doi: 10.3390/nu13113944 (PMC8623013; doi:10.3390/nu13113944)

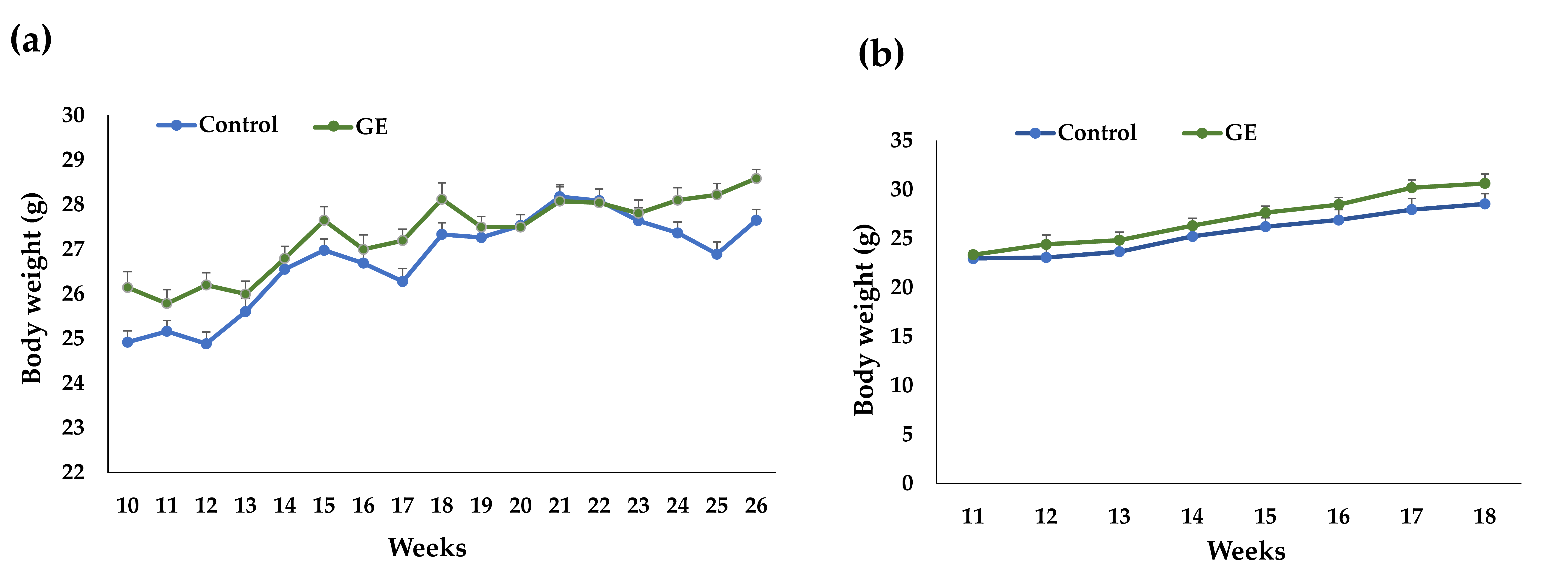

Supplement: Supplementary file 1 [file nutrients-13-03944-s001.zip › Figure S1.tif]

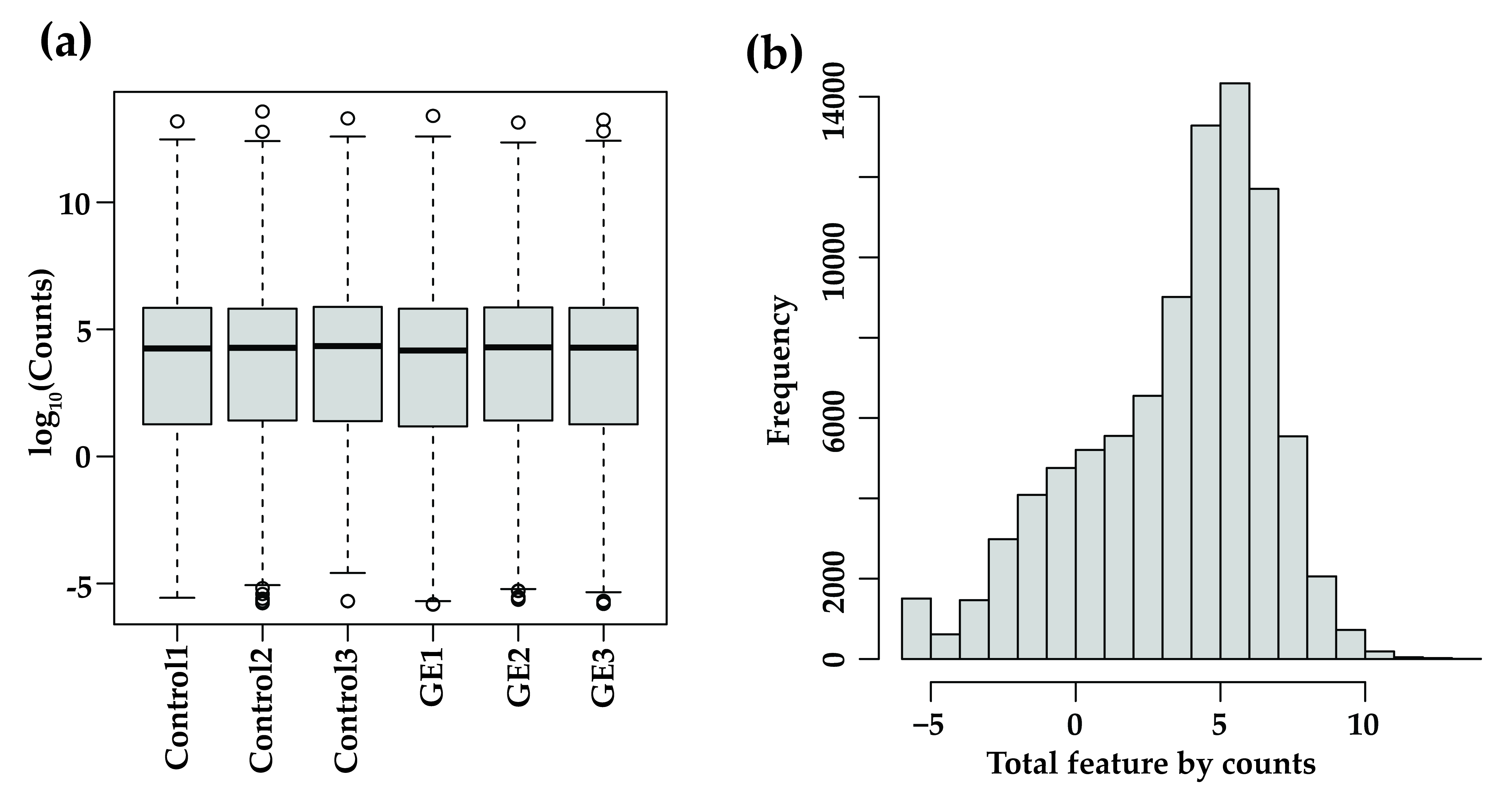

Supplement: Supplementary file 1 [file nutrients-13-03944-s001.zip › Figure S2.tif]
